# Re-Evaluation of the *Podosphaera tridactyla* Species Complex in Australia

**DOI:** 10.3390/jof7030171

**Published:** 2021-02-26

**Authors:** Reannon L. Smith, Tom W. May, Jatinder Kaur, Tim I. Sawbridge, Ross C. Mann, Ian G. Pascoe, Jacqueline Edwards

**Affiliations:** 1 Agriculture Victoria, Department of Jobs, Precincts and Regions, AgriBio Centre, Bundoora, VI 3083, Australia; jatinder.kaur@agriculture.vic.gov.au (J.K.); tim.sawbridge@agriculture.vic.gov.au (T.I.S.); ross.mann@agriculture.vic.gov.au (R.C.M.); jacky.edwards@agriculture.vic.gov.au (J.E.); 2 School of Applied Systems Biology, La Trobe University, Bundoora, VI 3083, Australia; 3 Royal Botanic Gardens Victoria, Melbourne, VI 3004, Australia; tom.may@rbg.vic.gov.au; 4 30 Beach Road, Rhyll, VI 3923, Australia; pascoeig@bigpond.net.au

**Keywords:** stone fruit, powdery mildew fungi, *Prunus*, *Podosphaera ampla*, *Podosphaera cunningtonii*

## Abstract

The *Podosphaera tridactyla* species complex is highly variable morphologically and causes powdery mildew on a wide range of *Prunus* species, including stone fruit. A taxonomic revision of the *Po. tridactyla* species complex in 2020 identified 12 species, seven of which were newly characterised. In order to clarify which species of this complex are present in Australia, next generation sequencing was used to isolate the fungal ITS+28S and host *matK* chloroplast gene regions from 56 powdery mildew specimens of stone fruit and ornamental *Prunus* species accessioned as *Po. tridactyla* or *Oidium* sp. in Australian reference collections. The specimens were collected in Australia, Switzerland, Italy and Korea and were collected from 1953 to 2018. Host species were confirmed using *matK* phylogenetic analysis, which identified that four had been misidentified as *Prunus* but were actually *Malus* *prunifolia*. *Podosphaera* species were identified using ITS+28S phylogenetic analysis, recognising three *Podosphaera* species on stone fruit and related ornamental *Prunus* hosts in Australia. These were *Po.* *pannosa*, the rose powdery mildew, and two species in the *Po. tridactyla* species complex: *Po. ampla*, which was the predominant species, and a previously unidentified species from peach, which we describe here as *Po. cunningtonii.*

## 1. Introduction 

Stone fruit such as peaches (*Prunus persica*), cherries (*Pr. avium*), apricots (*Pr. armeniaca*), plums (*Pr. domestica, Pr. salicina* and *Pr. cerasifera*) and almonds (*Pr. dulcis*) belong to the genus *Prunus* (Rosaceae), which contains approximately 250 species distributed across temperate regions worldwide [1]. In Australia, there are only two native *Prunus* species, *Pr. brachystachya* and *Pr. turneriana*; both are in the subgenus *Cerasus* and found in tropical rainforests of north east Australia. A further 15 species of *Prunus* have been introduced to Australia as horticultural crops and for use in gardens [2]. On the basis of phylogenetic analysis of multiple genes, the genus is subdivided into three subgenera: *Cerasus* (cherries), *Padus* (bird cherries, including species formerly placed in *Laurocerasus*) and *Prunus* (plums) and the latter subgenus is further subdivided into seven sections: *Amygdalus*, *Armeniaca*, *Emplectocladus*, *Microcerasus*, *Persicae*, *Prunocerasus* and *Prunus* [3]. The three subgenera are also distinguished on morphology according to inflorescence structure, where subgenus *Cerasus* has corymbose inflorescences, *Padus* has racemose inflorescences and *Prunus* has solitary inflorescences [4].

The Australian stone fruit industry was established in the late 1800s by European and Chinese settlers who introduced apricot, peach, nectarine and plums that were in cultivation across Asia, Europe and the USA. [5]. By 2017-18, the Australian stone fruit industry (comprising apricots, nectarines, peaches and plums) produced 153,148 tons (t) of fruit with a wholesale value of AUD 391.7 M. [6]. The 2017-18 net stone fruit supply was divided into export (17,769 t), processing (31,790 t) and domestic fresh supply (106,684 t). The export market is predominantly China, Indonesia, Singapore, Saudi Arabia and United Arab Emirates. Stone fruit is grown in temperate regions of all states of Australia, with Victoria producing the majority (108,197 t). Peaches and nectarines are the dominant crop (88,787 t), followed by plums (15,099 t) and apricots (4311 t) [6]. 

Stone fruit are affected by several powdery mildew fungi, including *Podosphaera tridactyla*, which is known to be highly variable morphologically, with a wide host range among *Prunus* species [7]. Powdery mildew infects leaves and stems, reducing the plant’s photosynthetic capabilities and fruit production [8]. Severe infection causes cupping and malformation of the leaves and infected fruit, resulting in further crop losses [9]. 

In 2005, Cunnington and co-workers investigated genetic variation within *Po. Tridactyla*, studying specimens from Australia, South Korea and Switzerland, using restriction fragment length polymorphism (RFLP) and rDNA ITS (internal transcribed spacer) sequence analyses [10]. RFLP analysis divided the specimens into six groups, with four of the groups (1–4) differing by a single restriction enzyme pattern. Based on the ITS sequence analysis, there were three well-supported clades. Clade 1 contained RFLP Group 5 from hosts including an unidentified *Prunus* sp., *Pr. cerasifera* and *Pr. armeniaca* (all belonging to *Prunus* subgenus *Prunus*). Clade 2 contained RFLP Groups 1–4 from *Pr. persica*, *Pr. japonica*, *Pr. padus*, *Pr. laurocerasus* and *Pr. lusitanica* (*Prunus* subgenera *Cerasus*, *Padus* and *Prunus*). Clade 3 contained RFLP Group 6 from an unknown *Prunus* sp. and *Pr. apetala* (*Prunus* subgenus *Cerasus*). The clade on hosts from *Prunus* subgenera *Cerasus*, *Padus* and *Prunus* represented *Po. tridactyla* in the strict sense, originally described from *Pr. padus*, while the other two taxa were undescribed species morphologically indistinguishable from *Po. tridactyla* in the strict sense.

In their taxonomic revision of Erysiphales, Braun and Cook [7] treated *Po. tridactyla* as a species complex, with no clear morphological delimitation between “typical” collections of *Po. tridactyla* and deviating forms. They did recognise *Podosphaera longiseta* as a distinct species within the *Po. tridactyla* species complex, but concluded that further molecular, biological and morphotaxonomic studies were required to fully recognise species diversity within the species complex. 

Meeboon et al. [11] published a wide ranging morphological and molecular taxonomic revision of powdery mildew fungi on *Prunus*, examining 30 specimens from 16 hosts and five countries across Asia and Europe. They characterised the *Po. tridactyla* species complex as comprising 12 species, of which seven were newly described: *Po. ampla*, *Po. pruni*-*avium*, *Po. pruni*-*cerasoidis*, *Po. prunigena*, *Po. pruni*-*japonicae*, *Po. pruni*-*lusitanicae* and *Po. prunina*. Additionally belonging to the complex were *Po. tridactyla* in the strict sense, *Po. longiseta* and *Po. salatai* and the two undescribed *Podosphaera* species from Australia. These two undescribed species formed distinct lineages but could not be described as the authors did not have access to the physical specimens, only DNA sequence data from Cunnington et al. [10]. Additionally, Meeboon et al. [11] identified host specificity at the subgenus level in *Prunus*, suggesting a degree of coevolution between species of the *Po. tridactyla* complex and their hosts. The radiation of *Prunus* subgenera identified by Chin et al. [4] conforms with the divergence of *Po. tridactyla* complex species across Eurasia, for example, *Po. tridactyla* in the strict sense is a European species found on hosts within *Prunus* subgenus *Padus* and *Po. ampla* is of Asian origin, infecting hosts within the *Prunus* subgenus *Prunus* [11].

The objective of the current study was to clarify which species of the *Po. tridactyla* complex are present in Australia, utilising the next generation sequencing (NGS) methods developed by Smith et al. [12], based on a re-examination of powdery mildew collections from horticultural *Prunus* species and closely related ornamental *Prunus* species in Australia held in Australian plant pathogen reference collections. 

## 2. Methods 

All powdery mildew collections identified as *Podosphaera tridactyla* or *Oidium* sp. on *Prunus* hosts were obtained from the three major Australian plant pathogen reference collections (Queensland Plant Pathogen Herbarium (BRIP—two collections), New South Wales Plant Pathology Biosecurity Collections (DAR—32 collections) and Victorian Plant Pathogen Herbarium (VPRI—125 collections)). Collections in BRIP and DAR were all from Australia, while those in VPRI were from Australia, Switzerland, Italy and Korea. All collections were inspected for DNA extraction suitability as described by Smith et al. [12], based on number of leaves in the packet, level of powdery mildew infection present on the leaves and if the specimens were glued to mounting paper. Using these criteria, 58 collections contained specimens that were suitable for DNA extraction (Table 1). 

### 2.1. Fungal Sampling and Morphological Characterisation

Powdery mildew conidia, mycelia and chasmothecia were collected from the specimens by using a 6 mm leaf punch or scraping with a blade. Sampling was completed under clean room conditions to minimise contamination with modern DNA.

The VPRI specimens collected between 1993 and 1995 were morphologically examined while fresh at the time of collection. The hyphae, conidiophores and conidia were collected off the leaf surface with clear cellotape, which was then mounted on a microscope slide with lactofuchsin mountant and observed using light microscopy. Lactofuchsin preparation was as follows: lactic acid 20 g, glycerol 40 g, water (H_2_O) 20 mL and acid fuchsin 0.1 g; this was adapted from the Johnson and Booth [13] lactophenol mountant recipe minus the phenol. Germination patterns were studied on host tissue by pressing a sporulating colony onto a fresh, healthy leaf and incubating the leaf in a petri dish with moist filter paper, then examined by cellotape strip after 24 and 48 h. Reference collection specimens collected between 1977 and 1992 were rehydrated before examination, as described by Shin [14] and Shin and La [15], by placing a small section of infected leaf upside down in a drop of lactic acid on a microscope slide, then placing a flame underneath to boil. Once boiled, the rehydrated fungal material was scraped off the leaf surface and mounted in lactic acid for examination by light microscopy [14,15]. 

Morphological characters were recorded for both fresh and reference collection specimens as follows: mycelium growth pattern and hyphal structure, mycelial appressoria shape, conidiophore characteristics (length, shape, position of basal septum), conidial chain edge line, conidial size and shape, presence or absence of fibrosin bodies and germination characters. 

### 2.2. DNA Extraction and Quantification

DNA was extracted following the E.Z.N.A.^®^ Forensic DNA protocol [12]. The DNA extraction initial incubation step was increased to one hour and included a repeated final elution step (50 µL of elution buffer were added to the filter column with a 5 min incubation for a final volume of 100 µL). A NanoDrop 2000™ (Thermo Fisher Scientific, Waltham, Massachusetts) was used to assess DNA quality using the 260/280 nm absorbency ratio (1.8–1.9). DNA concentrations were quantified using two methods: Quantus™ fluorometer (Promega) and Agilent Tapestation^®^ electrophoresis (Agilent Technologies, Santa Clara, CA, USA).

### 2.3. Library Preparation and Sequencing 

Library preparation was as described in Smith et al. [12]. Libraries comprising VPRI DNA were paired-end sequenced on the Illumina HiSeq 3000 platform (San Diego, CA, USA). Libraries comprising BRIP and DAR DNA were paired-end sequenced on the Illumina MiSeq platform, due to sequencing platform accessibility constraints at the time.

### 2.4. Sequence Analysis 

Sequence reads were assigned to each sample based on their indices. Raw DNA-Seq files were filtered using the Nuclear program to trim adapters and retain only the sequences above the cut-off value of 100 bp read length and 20 mismatches. The library preparations were expected to contain *Podosphaera* DNA and plant host DNA, as well as DNA from microbes present on the leaf surface at the time of collection. Therefore, the filtered, high-quality (HQ) reads were mapped as paired-end reads to *Podosphaera* and plant host reference sequences. For *Podosphaera*, the reference sequences used were the internal transcribed spacer ITS (ITS1-5.8S-ITS2) and 28S rDNA regions of *Po. ampla* (GenBank accession MK530453), *Po. cerasi* (KX826855), *Po. clandestina* (KY660805), *Po. leucotricha* (KX842350), *Po. longiseta* (MK530459), *Po. pannosa* (KX842349), *Po. prunigena* (AB936275), *Po. prunina* (MK530442), *Po. pruni*-*avium* (MK530457, KP641982), *Po. pruni*-*cerasoides* (MK530448), *Po. pruni*-*japonicae* (MK530455), *Po. pruni*-*lusitanica* (KP641993), *Po. salatai* (AB525929), *Po*. sp (AY833653) and *Po. tridactyla s*. *str*. (MK530462). The plant host was identified by mapping to *matK* chloroplast plastid regions. Reference sequences were those identified by Chin et al. [4] or taken directly from GenBank. The *matK* accessions from Chin et al. [4] were: *Pr. armeniaca* (HQ235100), *Pr. cerasifera* (HQ619834), *Prunus domestica* (HQ235146), *Pr. laurocerasus* (HQ235181), *Pr. mahaleb* (HQ235184), *Pr. padus* (HQ235216), *Pr. persica* (HQ235409), *Pr. salicina* (HQ235252), and the others from GenBank were *Pr. yedoensis* (GQ248191) and *Malus prunifolia* (JQ391019). The sequence mapping was performed by the program Nuclear, generating reference-initiated sequence alignments to be viewed in Vision software (version 3.3.6 Gydle Inc. Bioinformatics Service, Québec City, Québec; http://www/gydle.com, accessed on 3 February 2021). In Vision, ITS+28S and *mat*K sequences were edited to incorporate single nucleotide polymorphisms (SNPs) and indels which related to each individual specimen’s sequence data. A mapping threshold was determined by a minimum of 5x coverage of the complete reference sequence and sequence files which did not meet this criterion were excluded. Mapping success was determined from the Vision images of each sequence file by calculating the total number of aligned DNA sequence reads and converting it into a percentage from the total number of HQ reads. The mapped ITS+28S and *mat*K sequences generated for this study were exported from Vision for identification to species by BLASTn and phylogenetic analysis.

### 2.5. Phylogenetic Analysis 

**Figure 1:**
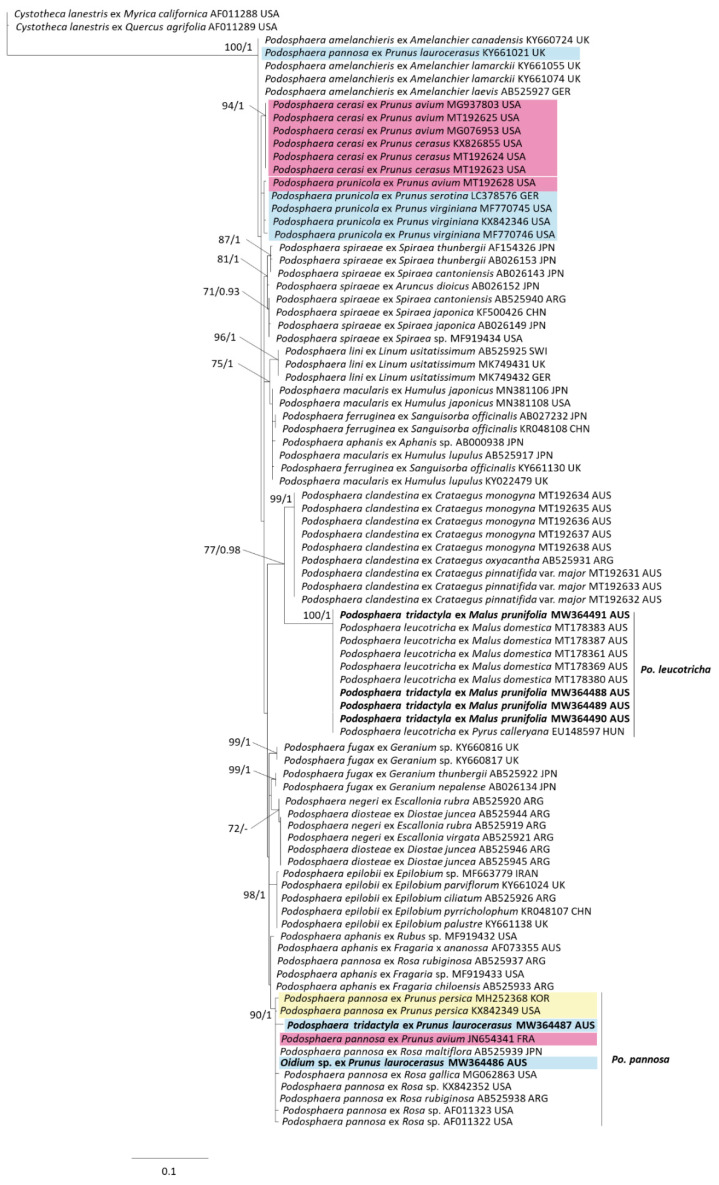
Maximum likelihood phylogenetic analysis of nuclear rDNA ITS (ITS1-5.8-ITS2) sequences for *Podosphaera pannosa* and *Po. leucotricha* and closely related species. Branch support values for maximum likelihood and Bayesian inference analyses are shown when >70% and 0.85, respectively. Sequences generated in this study are shown in bold with names as originally listed in reference collections. Label colours represent *Prunus* subgenera; *Cerasus* is pink, *Padus* is blue and *Prunus* is yellow.

**Figure 2:**
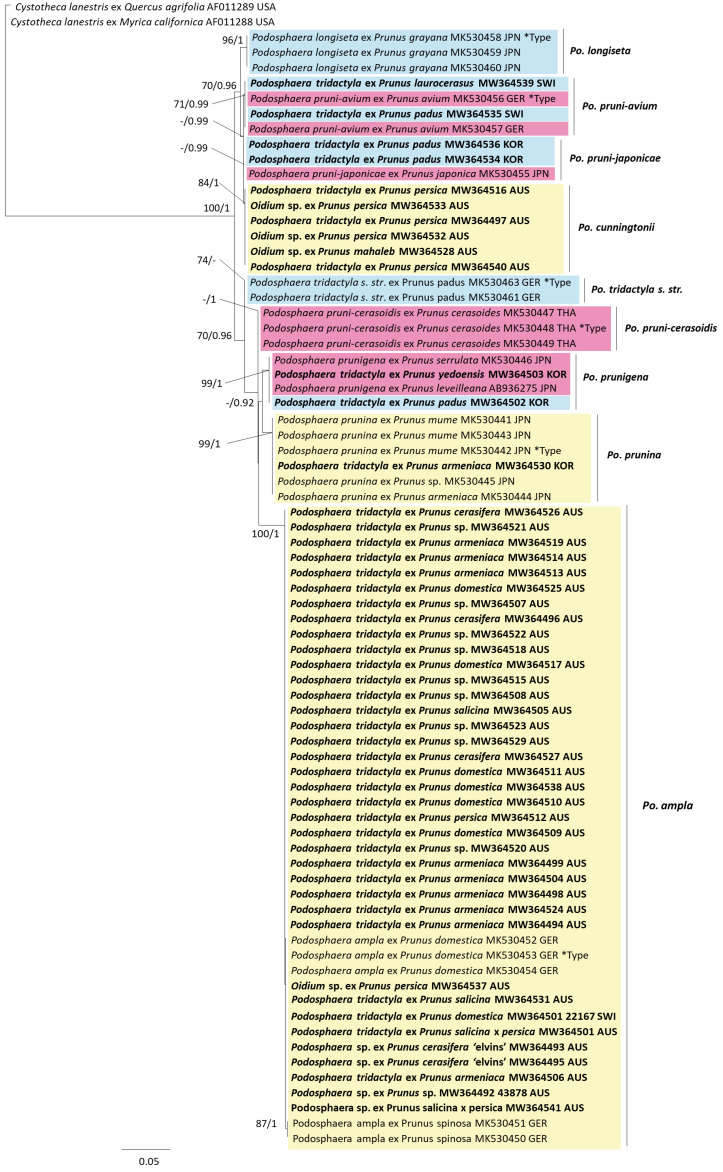
Maximum likelihood phylogenetic analysis of nuclear rDNA ITS1-5.8S-ITS2 and 28S sequences for the *Po. tridactyla* species complex and closely related species. Branch support values for maximum likelihood and Bayesian inference analyses are shown when >70% and 0.85, respectively. *Type indicates sequences obtained from isotype or holotype specimen for that species. Sequences generated in this study are shown in bold with names as originally listed in reference collections. Colours represent *Prunus* subgenera; *Cerasus* is pink, *Padus* is blue and *Prunus* is yellow.

Based on preliminary phylogenetic analysis, two data sets were generated for the sequences originally identified as *Po. tridactyla*. The first data set (Figure 1) was constructed for ITS (rDNA ITS1-5.8S-ITS2) sequences from VPRI *Po. tridactyla* specimens reidentified as *Po. leucotricha* and *Po. pannosa*, which included sequences from closely related *Podosphaera*. The second data set contained ITS+28S (rDNA ITS1-5.8S-ITS2 + rDNA 28S large subunit) sequences from the *Po. tridactyla* complex (Figure 2). Both data sets included sequences generated from this study and sequences of *Podosphaera* obtained from GenBank that were selected from published studies [11,16,17] and NCBI searches of *Podosphaera* spp. ITS and ITS+28S sequences. Phylogenetic analyses of these two data sets used *Cystotheca lanestris* (GenBank accessions AF011289 and AF011288) as the outgroup taxon, in line with Meeboon et al. [11]. The phylogenetic analysis of host *matK* sequences included sequences from *Prunus* species subgenera *Cerasus*, *Padus* and *Prunus* obtained from Chin et al. [4] and the outgroup was *Oemleria cerasiformis* (*matK* AF288110). Alignments were generated in Geneious Prime using the Muscle 3.8.425 [18,19] alignment tool with suggested settings followed by manual refinement. Phylogenetic trees were obtained from the aligned sequence data by maximum likelihood (ML) and Bayesian inference (BI) methods. ML analysis was performed in PhyML (3.3.20180621) [20] with the general time-reversible (GTR) substitution model with optimization for topology/length/rate with the proportion of invariable sites set at 0 and number of substitution rate categories at 4. The bootstrap analysis was set at 1000 replications with the stepwise addition option set as simple. BI analysis was completed in MrBayes (3.2.6) [21] using two runs with four chains each under the GTR model and run assuming a gamma distribution variation. Four heated chains and a single cold chain were used in all Markov chain Monte Carlo (MCMC) analyses, which were run for 1,100,000 generations and sampled one tree every 200 generations. Burn-in length was set at 100,000. 

To identify characteristic bases for sets of closely related species, summaries of variable base positions were generated for (1) *Po. ampla* and sister taxa and (2) species closely related to *Po. pruni*-*avium*. For each set of sequences, outgroup sequences were removed, and the sequences of interest were realigned. Masking of the amended alignments by removing sites containing identical bases, leaving only sites with variable bases (including gaps), was performed by the Mask Alignment tool in Geneious Prime. In addition, positions where only one sequence within a species varied from the consensus were removed manually in Geneious Prime. Base position numbers of the variable sites were manually edited in Microsoft PowerPoint to reflect base positions in the original alignment.

## 3. Results 

### 3.1. Sequencing

DNA from 56 of the 58 collections were successfully mapped to reference sequences of the powdery mildew (*Podosphaera*) ITS+28S region and host plant chloroplast gene *matK*. The exceptions were DAR 35281 and DAR 64667, which failed to meet the mapping threshold. The ITS+28S and *matK* sequences generated from this study were confirmed as belonging to *Podosphaera* and *Prunus*, respectively, using BLASTn. The BLASTn analysis of the powdery mildews detected six collections which did not match with species of the *Po. tridactyla* complex and matched to either *Po. pannosa* or *Po. leucotricha*. These six sequences were placed in a data set with other sequences identified at *Po. leucotricha* and *Po. pannosa* as well as closely related *Podosphaera* species, as they are not part of the *Po. tridactyla* species complex. The remaining ITS+28S sequences returned BLASTn percentage identities of at least 98% and up to 100% (E values were 0.0) with members of the *Po. tridactyla* species complex. 

### 3.2. Phylogeny

Phylogenetic analysis of the first data set compared ITS sequences of the six collections reidentified as *Po. pannosa* and *Po. leucotricha* with closely matched *Podosphaera* from GenBank and published sources [16,17] to total 88 sequences of 508 base pairs (Figure 1). A well-supported clade (bootstrap (BS) support 90 and posterior probability (BI) 1) consisted of nine sequences identified as *Po. pannosa* along with sequences from BRIP 8232 (originally identified as *Po. tridactyla*) and DAR 12478 (originally identified as *Oidium* sp.) Another well-supported clade (BS 100, BI 1) consisted of seven sequences identified as *Po. leucotricha* along with sequences from VPRI 12495, 18514, 18452 and 20687 (all originally identified as *Po. tridactyla*) (Figure 1).

Phylogenetic analysis of the second data set, comprising 75 ITS+28S sequences of 1251 base pairs, showed that collections from Australia represented two species of the *Po. tridactyla* species complex: *Po. ampla* on *Prunus* subgenus *Prunus* hosts and a previously undescribed *Podosphaera* species on *Prunus persica* and *Pr. mahaleb* (from *Prunus* subgenus *Prunus* and *Cerasus* (Figure 2)). A well-supported clade (BS 100, BI 1) consisted of five sequences from collections identified as *Po. ampla*, all from Germany, along with 37 sequences from Australian collections previously identified as *Po. tridactyla* and *Oidium* sp. (BRIP 15118). Within this clade, there was a moderately supported BI 0.85 subclade containing the *Po. ampla* reference sequences from Germany and nine sequences from Australian collections (Figure 2). Within this subclade, two of the collections from Germany (MK530450 and MK530451) fell into a further subclade (BS 87, BI 1). Additional VPRI collections from outside Australia fell within four of the established species: *Po. prunigena* (VPRI 20231 and 20491, South Korea), *Po. prunina* (VPRI 41641, South Korea), *Po. pruni*-*japonicae* (VPRI 20233 and 20490 South Korea) and *Po. pruni*-*avium* (VPRI 22156, Switzerland and VPRI 22159, Switzerland) (Figure 2) (Table 2).

A summary of variable bases for the *Po. ampla* sequences shows that the subclade containing the *Po. ampla* sequences from Germany, when compared to the remaining members of the clade, exhibits a one base pair difference at position 445 where a T is present instead of a C (Figure S1). Sequences MK530450 and MK530451 from Germany have two additional base changes at positions 813 (G instead of T) and 816 (A instead of G) (Figure S1). Sequence AF154321 displayed identical base pairs to other *Po. ampla* sequences, although it was significantly shorter at 479 bp in length. Sequence AY833656 was also shorter in length (503bp) but included three variants at positions 545 (A instead of G), 546 (T instead of A) and 550 (A instead of G). 

Separate analyses of alignments of each of ITS (ITS1-5.8S-ITS2), including 134 sequences covering 486 base pairs, and of 28S (large subunit) including 73 sequences of 808 base pairs, formed the same overall structure regarding all major clades. All Australian sequences generated from this study fell into two well-supported clades (*Po. ampla* and the unknown species of *Podosphaera*). Support values for the *Po. ampla* clade, including the Australian sequences, were as follows: ITS (BS 80, BI 1) and 28S (BS 100, BI 1) (Figures S2 and S3). In the ITS tree, two Australian sequences (AY833656 and AF154321) published by Cunnington et al. [10] and labelled as *Podosphaera* sp. by Meeboon et al. [11] fell into the *Po. ampla* clade. 

Eight Australian *Podosphaera* sequences on *Pr. persica* and *Pr. mahaleb* hosts, including two Australian sequences (AY833651 and AY833653) published by Cunnington et al. [10] and also labelled as *Podosphaera* sp. by Meeboon et al. [11], formed an independent lineage from other species of the *Po. tridactyla* complex. In the ITS+28S phylogeny, support for this new *Podosphaera* species is high (BS 84 and BI 1) (Figure 2). The individual ITS and 28S phylogenies also have strong support for this clade: ITS (BS 87, BI 1) and 28S (BS -, BI 0.98) (Figures S2 and S3). A summary of variable bases for the novel *Podosphaera* species shows five base-pair positions in 1234 characters at which there are differences from the two sister taxa, with differences at three positions compared to *Po. pruni*-*avium* and two positions compared to *Po. pruni*-*japonicae* (Figure S4). The new sequence generated for VPRI 19591 by next generation sequencing in the current study was taken from the same specimen that was used to generate sequence AY833653 by Sanger sequencing in 2005; the two sequences are identical. 

### 3.3. Fungus–Host Relationships

The ITS+28S phylogeny of the fungi showed several sequences from hosts belonging to different *Prunus* subgenera compared to the hosts detected by Meeboon et al. [11] (Figure 2). Three species, *Po. pruni*-*avium, Po. pruni*-*japonicae* and *Po. prunigena*, were all originally described from *Prunus* hosts within the subgenus *Cerasus*, but in the ITS+28S phylogeny, the clades for these species included VPRI 22159, VPRI 20490, VPRI 20233 and VPRI 20491 on *Pr. padus*, which is in subgenus *Padus*. The *Podosphaera pruni*-*japonicae* clade in the ITS tree also included sequences from fungi collections on *Prunus davidiana* (subgenus *Prunus*) (Figure S2). The ITS tree includes additional fungi sequences from GenBank, in which *Po. pruni*-*avium* forms two separate lineages; the first lineage consists of collections on both *Pr*. subgenus *Cerasus* and *Padus* and the second lineage has collections only on *Pr*. subgenus *Cerasus* (Figure S2). The clade consisting of collections of the undescribed powdery mildew on peach, *Prunus persica* (subgenus *Prunus*), also included a sequence from a collection on *Pr. mahaleb* (subgenus *Cerasus*). Both *Po. prunina* and *Po. ampla* clades contained powdery mildew sequences generated from *Prunus* hosts within subgenus *Prunus* in all three phylogenetic analyses.

### 3.4. Plant Host Phylogeny

**Figure 3:**
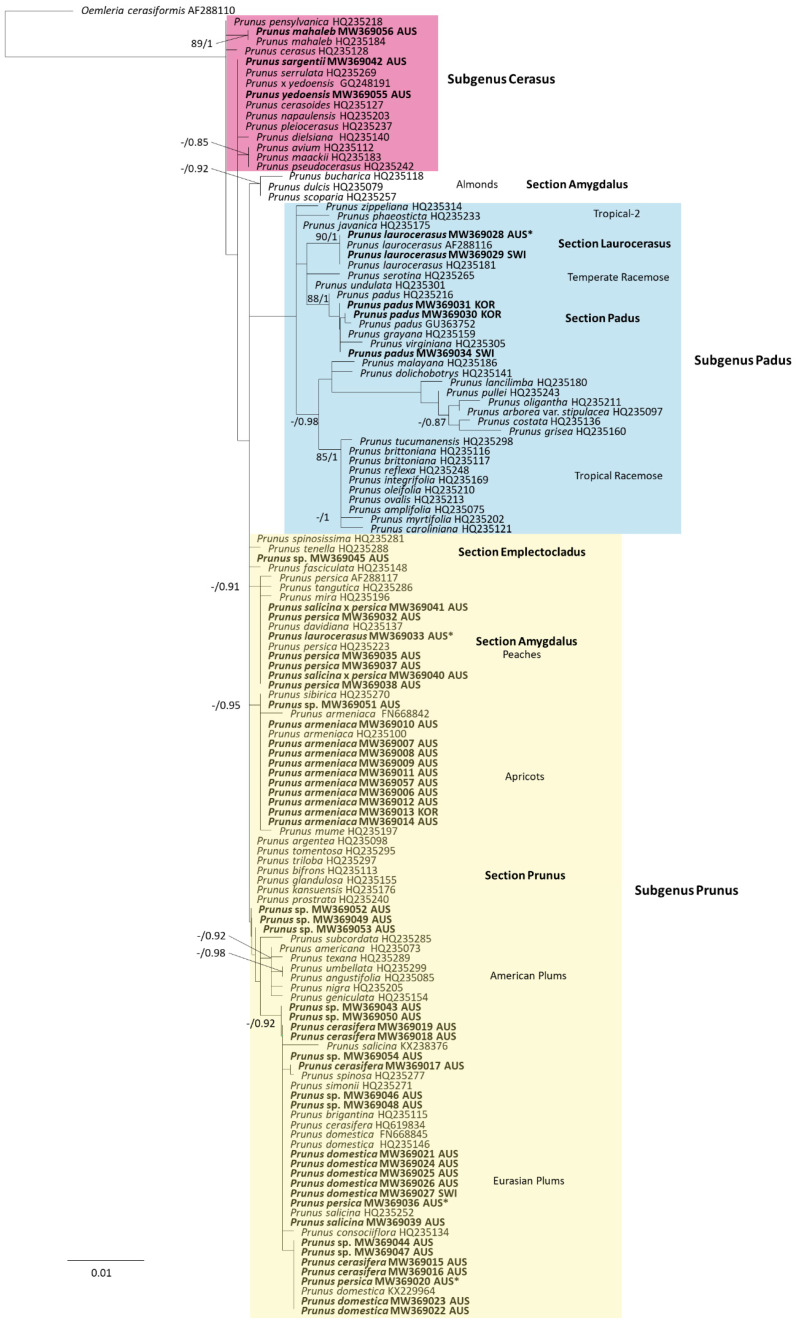
Maximum likelihood phylogenetic analysis of *matK* chloroplast gene sequences for host *Prunus* species. Branch support values for maximum likelihood (bootstrap (BS)) and Bayesian inference (BI) analyses are shown when >70% and 0.85, respectively. Colours represent *Prunus* subgenera; *Cerasus* is red, *Padus* is purple and *Prunus* is green. Sequences generated from this study are in bold and sequence names as deposited in reference collections. * Indicates a host that was originally misidentified (under original name).

There were nine different *Prunus* species originally listed as the plant hosts for Australian *Po. tridactyla* complex collections (*Pr. armeniaca*, *Pr. cerasifera*, *Pr. domestica*, *Pr. laurocerasus*, *Pr. mahaleb*, *Pr. padus*, *Pr. persica*, *Pr. salicina* and *Pr. yedoensis*). The plant host species were analysed in a *matK* phylogenetic analysis containing 133 sequences of 1288 base pairs, including representatives of the three subgenera of *Prunus* (Figure 3). 

The phylogeny forms three main clades which represented the *Prunus* subgenera *Cerasus* (clade 1), *Padus* (clade 2) and *Prunus* (clade 3). The *matK* sequences for DAR 28963, VPRI 20491 and VPRI 20231 fell into clades with sequences for *Pr. mahaleb* and *Pr. yedoensis* belonging to subgenus *Cerasus* within the *Padus* clade, VPRI 22156 and BRIP 8323 formed a well-supported clade (BS 90, BI 1) with sequences identified as *Pr. laurocerasus*. This is a host reidentification for BRIP 8323 which had *Pr. persica* listed on the specimen. Three VPRI sequences, 20490, 20233 and 22159, formed a clade with no BS support and BI support below the 0.85 threshold with *Pr. padus* sequences and close relatives such as *Pr. grayana* and *Pr. virginiana*. Within the subgenus *Prunus*, section *Amygdalus* formed a clade that was only supported by BI (0.91) containing sequences identified as *Pr. persica* along with sequences VPRI 19591, 20705, 20706, 22232 and 22233 (all originally identified as *Pr. persica* and *Pr. salicina* x *persica*) along with DAR 28962 and DAR 71638 (originally misidentified as *Pr. laurocerasus*). In section *Prunus,* the sequences identified as *Pr. armeniaca* formed a clade including eleven VPRI and DAR specimens identified as *Pr. armeniaca* with BI support of 0.95. The remaining VPRI sequences (originally identified as *Prunus* sp., *Pr. cerasifera*, *Pr. domestica*, *Pr. salicina* and *Pr. persica*) fell within a clade comprising sequences identified as Eurasian plums of sect. *Prunus* with high BI support (0.92) and no BS support. The Eurasian plums included *Pr. domestica*, *Pr. cerasifera* and *Pr. salicina*. There are minimal differences between these species within the *matK* gene region and therefore, for the purpose of identification, this group is referred to as the *Pr. domestica* group which includes the previously mentioned *Prunus* species as well as *Pr. brigantina*, *Pr. consociiflora*, *Pr. simonii* and *Pr. spinosa*. Additionally included in the clade of Eurasian plums were sequences from BRIP 15118 and VPRI 19868, which were both previously misidentified as *Pr. persica* (Figure 3). There were 14 specimens with plant hosts originally listed only as *Prunus* sp., in the *matK* phylogeny, these sequences fell within the clade of Eurasian plums in section *Prunus* (Table 2). 

There were four VPRI specimens, VPRI 12495, 18452, 18514 and 20687, which are labelled with plant hosts listed as *Prunus persica*, *Pr*. sp., *Pr. domestica* and *Pr*. sp., respectively. However, the *matK* sequences identified the hosts as *Malus prunifolia* (plum-leaf crab apple) and the associated powdery mildews were all re-identified as *Po. leucotricha*.

### 3.5. Morphological Characterisation

**Figure 4:**
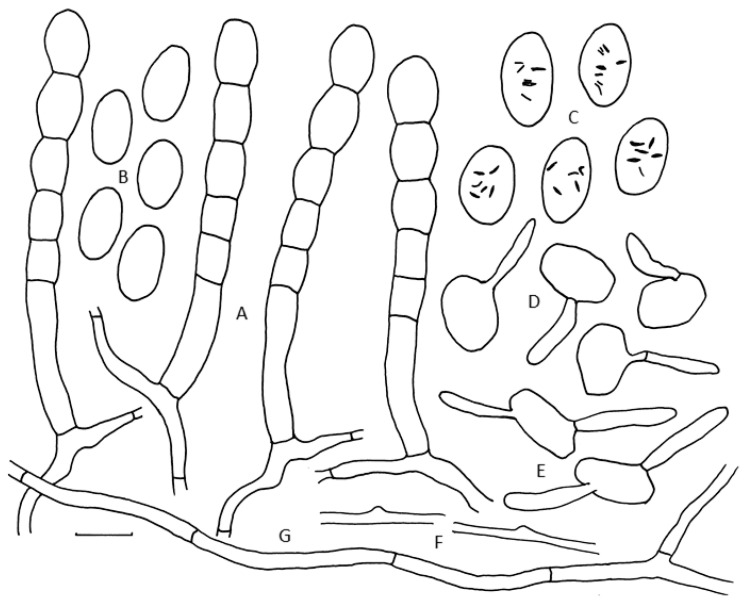
*Podosphaera cunningtonii* on *Prunus persica* morphology. (**A**) Conidiophores on Sellotape strip in lactic acid. (**B**) Conidia (mounted in lactofuchsin). (**C**) Conidia (mounted in water), showing fibrosin bodies. (**D**) Conidia germinated on host leaf for 24 h (mounted in lactofuchsin). (**E**) Conidia germinated on host leaf for 48 h (mounted in lactofuchsin). (**F**) Inconspicuous, nipple-shaped appressoria (mounted in lactofuchsin). (**G**) Hyphae on Sellotape strip (mounted in lactofuchsin). Scale bar = 20 µm. (All drawings from holotype VPRI 19591, except D & E from VPRI 20705).

The phylogenetic analysis showed a well-supported independent lineage of an undescribed species which is sister to *Po. pruni*-*avium* and *Po. pruni*-*japonicae*. No sexual morphs were found among material of this species. The morphologies of the sister taxa barely differ from *Po. tridactyla* in the strict sense and the asexual characters of this undescribed species on *Prunus persica* and *Pr. mahaleb* are the shorter conidiophore foot cells, crenate conidial chains and smaller conidia when compared to characters of the asexual morph of *Po. tridactyla*, which has long foot cells and ellipsoid(-doliform) conidia catenescent with crenate edge line (Figure 4). 

Based on the morphological and sequence differences, we propose the following new species.

***Podosphaera cunningtonii*** R.L. Smith, I. Pascoe, T.W. May and J. Edwards. Mycobank Number: MB838823. 

*Typification.* AUSTRALIA, VICTORIA: Burnley, isolated from *Prunus persica,* 28 October 1993, I. Pascoe (holotype VPRI 19591, dried culture; ITS+28S: MW364516). 

*Etymology*: the epithet commemorates Dr. James Cunnington, the first person to conduct molecular examination of this species.

Mycelium hyphae branched, thin walled, sinuous. Hyphal appressoria nipple shaped. Conidiophores straight, basal septum slightly displaced at junction, foot cells (30–) 43–60 × 8–11 µm, succeeded by 2–3 following cells, forming conidia in chains with crenate edge lines. Conidia ovoid(-doliform), 23–25 × 12–15 µm, fibrosin bodies present in water mounts, lacking in lactic acid mounts. Germination on host tissue at 24 h, producing single oblong-clavate, simple germ tubes 20–30 × 5–6 μm; at 48 h, producing 1–2 simple germ tubes 25–35 μm. No sexual morph observed. 

*Natural distribution*: currently unknown as isolates were collected in quarantine glasshouses in Australia. 

*Additional collections examined*: AUSTRALIA, VICTORIA: Knoxfield, isolated from *Pr. persica,* 7 March 1994, A. Sivapalan VPRI 19,868; isolated from *Pr. persica*, 27 September 1995, V. Beilharz VPRI 20,705; isolated from *Pr. persica*, 27 September 1995, V. Beilharz VPRI 20706; NEW SOUTH WALES: Rydalmere, isolated from *Pr. persica,* 3 June 1977, L. Penrose and J. Walker DAR 28,962; isolated from *Pr. mahaleb*, 3 June 1977, L. Penrose and J. Walker DAR 28,963; isolated from *Pr. persica*, 22 January 1996, G. Stovold DAR 71638 (sequence only, not examined morphologically).

## 4. Discussion 

This study re-evaluated reference collection specimens of *Po. tridactyla* and *Oidium* sp. on *Prunus* hosts and, through phylogenetic analysis, found seven different powdery mildew species and corrected misidentified plant hosts of eight specimens. The main species present on *Prunus* in Australia was *Po. ampla* and we also found that Australian collections of *Po. pannosa* and *Po. leucotricha* had been misidentified as *Po. tridactyla* and *Oidium* sp. in two reference collections. Finally, we characterised a new species in the *Po. tridactyla* complex which we name *Po. cunningtonii* from *Pr. persica* and *Pr. mahaleb* plant hosts. 

The phylogenetic analysis based on sequences derived from NGS shows that 37 of the 56 specimens fell within a clade alongside sequences identified as *Po. ampla* by Meeboon et al. [11]. Most Australian sequences assigned to *Po*. a*mpla* had a one base pair difference compared to the *Po. ampla* sequences analysed in previous studies. The revision of the *Po. tridactyla* species complex by Meeboon et al. [11] included a number of sequences containing several bases varying from the *Po. ampla* holotype MK530453 but were still included under *Po. ampla*. Therefore, most of the Australian powdery mildew fungi on stone fruit (*Prunus armeniaca*, *Pr. cerasifera*, *Pr. domestica* and *Pr. salicina*) formerly identified as *Po. tridactyla* are in fact *Po. ampla*.

The ITS phylogeny and 28S phylogenies also confirmed that most of the Australian sequences were *Po. ampla* and fell within a well-supported clade with *Po. ampla* sequences analysed by Meeboon et al. [11] (Figures S2 and S3). *Podosphaera tridactyla* sequences AF154321 and AY883656 generated by Cunnington et al. [10] were included in the ITS phylogeny and summary of variable sites and they both fell in with the other *Po. ampla* sequences, the only differences being that the sequence lengths were significantly shorter than the ITS+28S sequences which were generated by this study and Meeboon et al. [11] and AY883656 contained three variable base pairs towards the end of the sequence. We are confident in reidentifying these two sequences as *Po. ampla*.

The range of hosts for *Po. ampla* observed in this study included *Pr. cerasifera* and *Pr. domestica* as well as other species within subgenus *Prunus* section *Prunus* (Figure 3). In Meeboon et al. [11], *Po. ampla* was identified from *Pr. domestica* and *Pr. spinosa*, as well as from *Pr. armeniaca* and *Pr. cerasifera*. These *Prunus* hosts for *Po. ampla* were observed in this study as well as *Pr. salicina*, which is also within subgenus *Prunus* sect. *Prunus*. It would be expected in ideal conditions such as in a glasshouse for *Po. ampla* to infect a close relative of its listed hosts. 

The results of the three phylogenetic analyses from this study contradict host specificity at the subgenus level in *Prunus* for *Po. prunigena*, *Po. pruni*-*japonicae* and *Po. pruni*-*avium* species, as suggested by Meeboon et al. [11] because each fungus species clade contained *Prunus* hosts from subgenera *Cerasus* and *Padus*. Chin et al. [4] suggested that species within the subgenus *Cerasus* have a close alliance with some temperate racemose species found within subgenus *Padus*, such as *Pr. padus* and *Pr. laurocerasus*. This might offer an explanation as to the inclusion of supposedly different *Prunus* host subgenera in these host genera-specific powdery mildew fungi, as the remaining species of the *Po. tridactyla* complex are specific at the host subgenus level.

The remaining species which were identified from the Australian reference collections were: *Po. pannosa*, *Po. leucotricha*, *Po. pruni*-*japonicae*, *Po. pruni*-*avium*, *Po. prunina* and *Po. prunigena*. *Podosphaera pannosa* and *Po. leucotricha* were identified from Australian collections which had misidentified fungus and plant hosts recorded. The phylogeny showed four VPRI specimens were actually *Po. leucotricha* or the apple powdery mildew on plum-leaf crab apple (*M*. *prunifolia*) and two specimens from BRIP and DAR were *Po. pannosa*, which is more commonly seen on roses. The remaining *Po. tridactyla* complex species were identified from collections made in Switzerland and South Korea but held at VPRI.

While most Australian specimens were *Po. ampla*, six of the sequences newly generated by NGS were of an unknown species which did not match any identified sequences on GenBank. Cunnington et al. [10] and Meeboon et al. [11] both stated that two of the six sequences represented in their studies were an independent genetic lineage within the *Po. tridactyla* complex. In the Cunnington et al. [10] study, the two sequences AY833651 and AY833653 formed a clade which was sister to a clade comprising *Podosphaera* on *Pr. japonica*, which was renamed *Po. pruni*-*japonicae* by Meeboon et al. [11], within clade 2, which contained sequences of *Po. tridactyla* not on *Prunus* subgenus *Prunus*. In the Meeboon et al. [11] study, the phylogeny showed sequences AY833651 and AY833653 forming a separate clade sister to a clade comprising *Po. pruni*-*japonicae* and *Po. pruni*-*avium*. The results of the present phylogenetic study align with both Cunnington et al. [10] and Meeboon et al. [11], where the ITS+28S phylogeny shows that sequences of *Po. cunningtonii* including additional sequences generated in this study form a well-supported clade which is sister to a clade comprising *Po. pruni*-*japonica* and *Po. pruni-avium*.

A GenBank search for sequences of *Podosphaera* species on *Prunus persica* returned 26 sequences of two powdery mildew species, *Po. pannosa* and *Po. leucotricha* (peach rusty spot), which are both found on peach [22,23,24]. However, the sequences of this new species do not match either of these powdery mildew species, further confirming a previously undescribed powdery mildew. Braun and Cook [7] list three powdery mildew species with *Pr. persica* included in the host ranges, these are *Po. tridactyla* in the strict sense, *Po. prunicola* and *Po. pannosa*, which are quite separate to *Po. cunningtonii* in the phylogeny. 

At the time of the first collections of *Po. cunningtonii* in Victoria (mid-1990s), it was assumed that the quarantine glasshouse-grown *Pr. persica* had been infected by powdery mildew blowing into the glasshouse (Cunnington 2020 pers. comm.). However, in this study, we identified the same powdery mildew fungi on three specimens from New South Wales, two of which (DAR 28962 and DAR 28963) were also collected in quarantine glasshouses. The two specimens from quarantine glasshouses in N.S.W. are on two different hosts—*Pr. persica* (subgenus *Prunus*) and *Pr. mahaleb* (subgenus *Cerasus*). Notes in the specimen packet reported that the *Pr. mahaleb* seedlings were growing next to heavily powdery mildew-infected *Pr. persica* seedlings in the same glasshouse. The determiner, Dr. John Walker, noted at the time of collection (1977), “mildew development very light and feel that this is an adventitious development of mildew on this host from the heavily mildewed peach seedlings growing nearby”. We suggest that the primary host of this new powdery mildew is *Pr. persica* but under glasshouse conditions it can infect other *Prunus* species from different subgenera as accidental hosts. 

A third specimen of *Po. cunningtonii*, from N.S.W. (DAR 71638), was collected from *Pr. persica* on a private property in 1996, in a locality with several orchards that grew peach and other stone fruit in the near vicinity. This is the only report of *Po. cunningtonii* from outside of quarantine glasshouses and therefore we cannot confirm if it is established in Australia. Two specimens of powdery mildew on *Pr. persica* collected in 1991 from a quarantine facility in Alice Springs, Northern Territory, are lodged in the Northern Territory Plant Pathogen Collection (DNAP). Unfortunately, we were unaware of them at the time of commencement of the present study, so they were not included. At the time of collection, they were identified morphologically as *Po. pannosa* by determiner Dr. Jose Liberato. The two native *Prunus* species, *Pr. brachystachya* and *Pr. turneriana*, are only found in tropical rainforests in the far north of Queensland of Australia. Horticultural *Prunus* species, including stone fruit, are grown in the temperate regions of Australia further south [2,6]. Geographical distance and climatic differences would prevent the spread of a “native” *Prunus* powdery mildew fungus onto commercial stone fruit crops and to date there are no records of powdery mildew fungi infecting these native hosts. It is highly unlikely that *Po. cunningtonii* has a native area of distribution in the natural distribution of *Pr. persica* which originates from Northwest China. New peach varieties are imported into Australia as budwood and grown under quarantine prior to release. Potential explanations as to why *Po. cunningtonii* has not been observed more widely in Australia could be that infected plants would have not been released from quarantine. Another reason could be that the local climate in peach-growing regions in Australia is not climatically suitable for *Po. cunningtonii* development, whereas the quarantine glasshouse conditions provided an artificial environment for *Po. cunningtonii* growth [25,26]. 

Australia is believed to be a continent without native powdery mildew fungi, with accidental human-assisted powdery mildew introductions occurring post-European settlement on non-native agricultural, horticultural, ornamental and pasture plant species [27]. Further evidence for the lack of native powdery mildew fungi in Australia is the evolution of native plant species in long isolation from continents that now make up the northern hemisphere, which is where powdery mildew fungi appear to have originated and subsequently co-evolved with plant hosts [27]. Walker [28] investigated the distribution of plant-parasitic fungi across Australia and observed that most accounts of powdery mildew infection were on introduced monocot and dicot plant species and only a few on native plant species, with these infections occurring in artificial settings such as glasshouses or nurseries. After analysis of records of powdery mildew fungi from Australia, Kiss et al. [27] concluded that through European settlement, almost all of the agricultural and horticultural crops grown in Australia were recently introduced and with them powdery mildew. They also found that freshly collected powdery mildew specimens from Australia were phylogenetically similar to species known overseas and were collected mostly on imported plant hosts but were also identified from 13 native plant species [27].

Over the last 50 years, there have been changes in the way that specimens are collected, preserved, studied and recorded in reference collections [29]. These changes include increased use of molecular analysis for identification, publication of sequence data in online resources such as GenBank, digitisation of reference collection specimens and exposure via associated online catalogues. We detected numerous misidentified *Po. tridactyla* specimens and we also identified and described a previously unrecorded powdery mildew species, *Po. cunningtonii*. Outcomes such as these highlight the need to re-evaluate important plant pathogen groups held in reference collections, in particular unculturable pathogens and complexes of cryptic species [30]. Australia implements a high level of biosecurity and quarantine measures to prevent entry of unwanted pests and pathogens which can affect the Australian horticultural industry and trade (Hyde et al. 2010). In order to provide this pest and pathogen information, the specimen records must be up to date with current taxonomic classification and names in order to provide accurate species lists of pathogens currently present in Australia [31]. 

Through NGS applications, re-examination of selected powdery mildew specimens from Australian plant pathogen reference collections has demonstrated that one species, *Po. ampla*, is the dominant powdery mildew infecting stone fruit in Australia. In addition, the phylogenetic study confirmed the presence of *Po. pannosa* in Australia and allowed delimitation of a new powdery mildew species within the *Po. tridactyla* species complex, *Po. cunningtonii* on *Pr. persica*, for which we provide a morphological and molecular description. This study resolved powdery mildew species but also identified *Prunus* plant hosts and reclassified previously misidentified or incompletely identified hosts of specimens held in Australian plant pathogen reference collections. The information generated in this study will be used to update BRIP, DAR and VPRI powdery mildew specimens to provide accurate data for Australian biosecurity agencies. 

## Figures and Tables

**Table 1:** Collection details for the 58 powdery mildew collections on *Prunus* used in this study with fungus and plant host species as listed with collection and GenBank accession numbers. For hosts, abbreviations in brackets refer to divisions within *Prunus*: AM: subgenus *Prunus* section *Amygdalus*, CR: subgenus *Cerasus*, LR: subgenus *Padus* section *Laurocerasus*, PD: subgenus *Padus* section *Padus* and PR: subgenus *Prunus* section *Prunus*. *Po*. = *Podosphaera*, *Pr*. = *Prunus*.

				GenBank ITS+28S		GenBank *matK*
Collection Number	Date	Country	Fungus		Host	
BRIP 8323	1958	Australia	*Po. tridactyla*	MW364487	*Pr. persica* (AM)	MW369028
BRIP 15118	1986	Australia	*Oidium* sp.	MW364537	*Pr. persica* (AM)	MW369020
DAR 12478	1962	Australia	*Oidium* sp.	MW364486	*Pr. armeniaca* (PR)	MW369006
DAR 28962	1977	Australia	*Oidium* sp.	MW364533	*Pr. persica* (AM)	MW369032
DAR 28963	1977	Australia	*Oidium* sp.	MW364528	*Pr. mahaleb* (CR)	MW369056
DAR 35281	1980	Australia	*Oidium* sp.	*-*	*Pr. laurocerasus* (LR)	-
DAR 64667	1989	Australia	*Po. tridactyla*	*-*	*Pr. persica* (AM)	-
DAR 71638	1996	Australia	*Oidium* sp.	MW364532	*Pr. laurocerasus* (LR)	MW369033
VPRI 12495	1984	Australia	*Po. tridactyla*	MW364488	*Pr. persica* (AM)	MW369060
VPRI 18452	1992	Australia	*Po. tridactyla*	MW364489	*Prunus* sp.	MW369058
VPRI 18514	1992	Australia	*Po. tridactyla*	MW364490	*Pr. domestica* (PR)	MW369061
VPRI 18600	1992	Australia	*Po. tridactyla*	MW364521	*Prunus* sp.	MW369043
VPRI 18885	1993	Australia	*Po. tridactyla*	MW364522	*Prunus* sp.	MW369044
VPRI 19000	1993	Australia	*Po. tridactyla*	MW364523	*Prunus* sp.	MW369045
VPRI 19006	1993	Australia	*Po. tridactyla*	MW364529	*Prunus* sp.	MW369050
VPRI 19008	1993	Australia	*Po. tridactyla*	MW364524	*Pr. armeniaca* (PR)	MW369007
VPRI 19015	1993	Australia	*Po. tridactyla*	MW364538	*Pr. domestica* (PR)	MW369021
VPRI 19017	1993	Australia	*Po. tridactyla*	MW364525	*Pr. domestica* (PR)	MW369022
VPRI 19022	1993	Australia	*Po. tridactyla*	MW364520	*Prunus* sp.	MW369046
VPRI 19164	1993	Australia	*Po. tridactyla*	MW364519	*Pr. armeniaca* (PR)	MW369008
VPRI 19238	1993	Australia	*Po. tridactyla*	MW364526	*Pr. cerasifera* (PR)	MW369015
VPRI 19248	1993	Australia	*Po. tridactyla*	MW364518	*Prunus* sp.	MW369047
VPRI 19319	1993	Australia	*Po. tridactyla*	MW364517	*Pr. domestica* (PR)	MW369023
VPRI 19591	1993	Australia	*Po. tridactyla*	MW364516	*Pr. persica* (AM)	MW369035
VPRI 19788	1994	Australia	*Po. tridactyla*	MW364515	*Prunus* sp.	MW369048
VPRI 19837	1994	Australia	*Po. tridactyla*	MW364527	*Pr. cerasifera* (PR)	MW369016
VPRI 19864	1994	Australia	*Po. tridactyla*	MW364514	*Pr. armeniaca* (PR)	MW369009
VPRI 19865	1994	Australia	*Po. tridactyla*	MW364513	*Pr. armeniaca* (PR)	MW369010
VPRI 19868	1994	Australia	*Po. tridactyla*	MW364512	*Pr. persica* (AM)	MW369036
VPRI 19871	1994	Australia	*Po. tridactyla*	MW364511	*Pr. domestica* (PR)	MW369024
VPRI 19872	1994	Australia	*Po. tridactyla*	MW364510	*Pr. domestica* (PR)	MW369025
VPRI 19873	1994	Australia	*Po. tridactyla*	MW364509	*Pr. domestica* (PR)	MW369026
VPRI 20027	1994	Australia	*Po. tridactyla*	MW364508	*Prunus* sp.	MW369049
VPRI 20040	1994	Australia	*Po. tridactyla*	MW364507	*Prunus* sp.	MW369051
VPRI 20041	1994	Australia	*Po. tridactyla*	MW364506	*Prunus* sp.	MW369052
VPRI 20045	1994	Australia	*Po. tridactyla*	MW364505	*Pr. salicina* (PR)	MW369039
VPRI 20097	1994	Australia	*Po. tridactyla*	MW364504	*Pr. armeniaca* (PR)	MW369011
VPRI 20231	1990	South Korea	*Po. tridactyla*	MW364503	*Pr. yedoensis* (CR)	MW369055
VPRI 20233	1993	South Korea	*Po. tridactyla*	MW364534	*Pr. padus* (PD)	MW369030
VPRI 20490	1993	South Korea	*Po. tridactyla*	MW364536	*Pr. padus* (PD)	MW369031
VPRI 20491	1993	South Korea	*Po. tridactyla*	MW364502	*Pr. sargentii* (CR)	MW369042
VPRI 20514	1995	Australia	*Po. tridactyla*	MW364496	*Pr. cerasifera* (PR)	MW369017
VPRI 20687	1995	Australia	*Po. tridactyla*	MW364491	*Prunus* sp.	MW369059
VPRI 20705	1995	Australia	*Po. tridactyla*	MW364497	*Pr. persica* (AM)	MW369037
VPRI 20706	1995	Australia	*Po. tridactyla*	MW364540	*Pr. persica* (AM)	MW369038
VPRI 20797	1996	Australia	*Po. tridactyla*	MW364498	*Pr. armeniaca* (PR)	MW369057
VPRI 20811	1996	Australia	*Po. tridactyla*	MW364499	*Pr. armeniaca* (PR)	MW369012
VPRI 21717	1998	Australia	*Po. tridactyla*	MW364531	*Prunus* sp.	MW369053
VPRI 22156	1995	Switzerland	*Po. tridactyla*	MW364539	*Pr. laurocerasus* (LR)	MW369029
VPRI 22159	1994	Switzerland	*Po. tridactyla*	MW364535	*Pr. padus* (PD)	MW369034
VPRI 22167	1995	Switzerland	*Po. tridactyla*	MW364500	*Pr. domestica* (PR)	MW369027
VPRI 22232	2000	Australia	*Po. tridactyla*	MW364541	*Pr. salicina x persica* (PR/AM)	MW369040
VPRI 22233	2000	Australia	*Po. tridactyla*	MW364501	*Pr. salicina x persica* (PR/AM)	MW369041
VPRI 41641	2006	South Korea	*Po. tridactyla*	MW364530	*Pr. armeniaca* (PR)	MW369013
VPRI 43878	2018	Australia	*Podosphaera* sp.	MW364492	*Prunus* sp.	MW369054
VPRI 43879	2018	Australia	*Podosphaera* sp.	MW364493	*Pr. cerasifera “elvins”* (PR)	MW369018
VPRI 43880	2018	Australia	*Podosphaera* sp.	MW364495	*Pr. cerasifera “elvins”* (PR)	MW369019
VPRI 43881	2018	Australia	*Podosphaera* sp.	MW364494	*Pr. armeniaca* (PR)	MW369014

**Table 2:** Powdery mildew fungal specimens with updated fungus and host species names. These revised identifications are based on ITS+28S and chloroplast gene *matK* phylogenies, respectively. For hosts, abbreviations in brackets refer to divisions within *Prunus*: AM: subgenus *Prunus* section *Amygdalus*, CR: subgenus *Cerasus*, LR: subgenus *Padus* section *Laurocerasus*, PD: subgenus *Padus* section *Padus* and PR: subgenus *Prunus* section *Prunus*. The *Prunus domestica* group includes *Pr. domestica*, *Pr. cerasifera* and *Pr. salicina*, which are not readily distinguishable in the host *matK* phylogeny. * Indicates a host that was originally misidentified. *Po*. = *Podosphaera*, *Pr*. = *Prunus*.

Collection Number	Original Fungus	Reidentified Fungus	Original Host	Reidentified Host
BRIP 8323	*Po. tridactyla*	*Po. pannosa*	*Pr. persica* (AM) *	*Pr. laurocerasus* (LR)
BRIP 15118	*Oidium* sp.	*Po. ampla*	*Pr. persica* (AM) *	*Pr. domestica* group (PR)
DAR 12478	*Oidium* sp.	*Po. pannosa*	*Pr. armeniaca* (PR)	*Pr. armeniaca* (PR)
DAR 28962	*Oidium* sp.	*Po. cunningtonii*	*Pr. persica* (AM)	*Pr. persica* (AM)
DAR 28963	*Oidium* sp.	*Po. cunningtonii*	*Pr. mahaleb* (CR)	*Pr. mahaleb* (CR)
DAR 71638	*Oidium* sp.	*Po. cunningtonii*	*Pr. laurocerasus* (LR) *	*Pr. persica* (AM)
VPRI 12495	*Po. tridactyla*	*Po. leucotricha*	*Pr. persica* (AM) *	*Malus prunifolia*
VPRI 18452	*Oidium* sp.	*Po. leucotricha*	*Pr. laurocerasus* (LR) *	*M prunifolia*
VPRI 18514	*Po. tridactyla*	*Po. leucotricha*	*Pr. persica* (AM) *	*M. prunifolia*
VPRI 18600	*Po. tridactyla*	*Po. ampla*	*Prunus* sp.	*Pr. domestica* group (PR)
VPRI 18885	*Po. tridactyla*	*Po. ampla*	*Pr. domestica* (PR)	*Pr. domestica* group (PR)
VPRI 19000	*Po. tridactyla*	*Po. ampla*	*Prunus* sp.	*Pr. domestica* group (PR)
VPRI 19006	*Po. tridactyla*	*Po. ampla*	*Prunus* sp.	*Pr. domestica* group (PR)
VPRI 19008	*Po. tridactyla*	*Po. ampla*	*Prunus* sp.	*Pr. armeniaca* (PR)
VPRI 19015	*Po. tridactyla*	*Po. ampla*	*Prunus* sp.	*Pr. domestica* group (PR)
VPRI 19017	*Po. tridactyla*	*Po. ampla*	*Pr. armeniaca* (PR)	*Pr. domestica* group (PR)
VPRI 19022	*Po. tridactyla*	*Po. ampla*	*Pr. domestica* (PR)	*Pr. domestica* group (PR)
VPRI 19164	*Po. tridactyla*	*Po. ampla*	*Pr. domestica* (PR)	*Pr. armeniaca* (PR)
VPRI 19238	*Po. tridactyla*	*Po. ampla*	*Prunus* sp.	*Pr. domestica* group (PR)
VPRI 19248	*Po. tridactyla*	*Po. ampla*	*Pr. armeniaca* (PR)	*Pr. domestica* group (PR)
VPRI 19319	*Po. tridactyla*	*Po. ampla*	*Pr. cerasifera* (PR)	*Pr. domestica* group (PR)
VPRI 19591	*Po. tridactyla*	*Po. cunningtonii*	*Prunus* sp.	*Pr. persica* (AM)
VPRI 19788	*Po. tridactyla*	*Po. ampla*	*Pr. domestica* (PR)	*Pr. domestica* group (PR)
VPRI 19837	*Po. tridactyla*	*Po. ampla*	*Pr. persica* (AM)	*Pr. domestica* group (PR)
VPRI 19864	*Po. tridactyla*	*Po. ampla*	*Prunus* sp.	*Pr. armeniaca* (PR)
VPRI 19865	*Po. tridactyla*	*Po. ampla*	*Pr. cerasifera* (PR)	*Pr. armeniaca* (PR)
VPRI 19868	*Po. tridactyla*	*Po. ampla*	*Pr. armeniaca* (PR) *	*Pr. domestica* group (PR)
VPRI 19871	*Po. tridactyla*	*Po. ampla*	*Pr. armeniaca* (PR)	*Pr. domestica* group (PR)
VPRI 19872	*Po. tridactyla*	*Po. ampla*	*Pr. persica* (AM) *	*Pr. domestica* group (PR)*
VPRI 19873	*Po. tridactyla*	*Po. ampla*	*Pr. domestica* (PR)	*Pr. domestica* group (PR)
VPRI 20027	*Po. tridactyla*	*Po. ampla*	*Pr. domestica* (PR)	*Pr. domestica* group (PR)
VPRI 20040	*Po. tridactyla*	*Po. ampla*	*Pr. domestica* (PR)	*Pr. domestica* group (PR)
VPRI 20041	*Po. tridactyla*	*Po. ampla*	*Prunus* sp.	*Pr. domestica* group (PR)
VPRI 20045	*Po. tridactyla*	*Po. ampla*	*Prunus* sp.	*Pr. domestica* group (PR)
VPRI 20097	*Po. tridactyla*	*Po. ampla*	*Prunus* sp.	*Pr. domestica* group (PR)
VPRI 20231	*Po. tridactyla*	*Po. prunigena*	*Pr. salicina* (PR)	*Pr. yedoensis* (CR)
VPRI 20233	*Po. tridactyla*	*Po. pruni-japonicae*	*Pr. armeniaca* (PR)	*Pr. padus* (PD)
VPRI 20490	*Po. tridactyla*	*Po. pruni-japonicae*	*Pr. yedoensis* (CR)	*Pr. padus* (PD)
VPRI 20491	*Po. tridactyla*	*Po. prunigena*	*Pr. padus* (PD)	*Pr. sargentii* (CR)
VPRI 20514	*Po. tridactyla*	*Po. ampla*	*Pr. padus* (PD)	*Pr. domestica* group (PR)
VPRI 20687	*Po. tridactyla*	*Po. leucotricha*	*Pr. sargentii* (CR) *	*M. prunifolia*
VPRI 20705	*Po. tridactyla*	*Po. cunningtonii*	*Pr. cerasifera* (PR)	*Pr. persica* (AM)
VPRI 20706	*Po. tridactyla*	*Po. cunningtonii*	*Prunus* sp.	*Pr. persica* (AM)
VPRI 20797	*Po. tridactyla*	*Po. ampla*	*Pr. persica* (AM)	*Pr. armeniaca* (PR)
VPRI 20811	*Po. tridactyla*	*Po. ampla*	*Pr. armeniaca* (PR)	*Pr. armeniaca* (PR)
VPRI 21717	*Po. tridactyla*	*Po. ampla*	*Prunus* sp.	*Pr. domestica* group (PR)
VPRI 22156	*Po. tridactyla*	*Po. pruni-avium*	*Pr. laurocerasus* (LR)	*Pr. laurocerasus* (LR)
VPRI 22159	*Po. tridactyla*	*Po. pruni-avium*	*Pr. padus* (PD)	*Pr. padus* (PD)
VPRI 22167	*Po. tridactyla*	*Po. ampla*	*Pr. domestica* (PR)	*Pr. domestica* group (PR)
VPRI 22232	*Po. tridactyla*	*Po. ampla*	*Pr. salicina x persica* (PR/AM)	*Pr. persica* (AM)
VPRI 22233	*Po. tridactyla*	*Po. ampla*	*Pr. salicina x persica* (PR/AM)	*Pr. persica* (AM)
VPRI 41641	*Po. tridactyla*	*Po. prunina*	*Pr. armeniaca* (PR)	*Pr. armeniaca* (PR)
VPRI 43878	*Podosphaera* sp.	*Po. ampla*	*Prunus* sp.	*Pr. domestica* group (PR)
VPRI 43879	*Podosphaera* sp.	*Po. ampla*	*Pr. cerasifera “elvins”* (PR)	*Pr. domestica* group (PR)
VPRI 43880	*Podosphaera* sp.	*Po. ampla*	*Pr. cerasifera “elvins”* (PR)	*Pr. domestica* group (PR)
VPRI 43881	*Podosphaera* sp.	*Po. ampla*	*Pr. armeniaca* (PR)	*Pr. armeniaca* (PR)

## Data Availability

The data sets generated and analysed during the current study are available from the corresponding author on reasonable request.

## Supplementary Materials

The following are available online at https://www.mdpi.com/2309-608X/7/3/171/s1, Figure S1: Summary of variable sites for *Podosphaera ampla* and sister taxa *Po. prunina* and *Po. prunigena*, Figure S2: Maximum likelihood phylogenetic analysis of nuclear rDNA ITS1-5.8S-ITS2 sequences for the *Po. tridactyla* species complex and closely related species, Figure S3: Maximum likelihood phylogenetic analysis of nuclear rDNA 28S sequences for the *Po. tridactyla* species complex and closely related species, Figure S4: Summary of variable sites for *Po. cunningtonii* sequences compared to sister taxa *Podosphaera pruni-avium* and *Po. pruni-japonicae*.
