# Supplementary material for: Re-Evaluation of the Podosphaera tridactyla Species Complex in Australia
Source: J Fungi (Basel). 2021 Feb 26;7(3):171. doi: 10.3390/jof7030171 (PMC8025908; doi:10.3390/jof7030171)
Supplement: Supplementary file 1 [file jof-07-00171-s001.zip › Figure S1.docx]

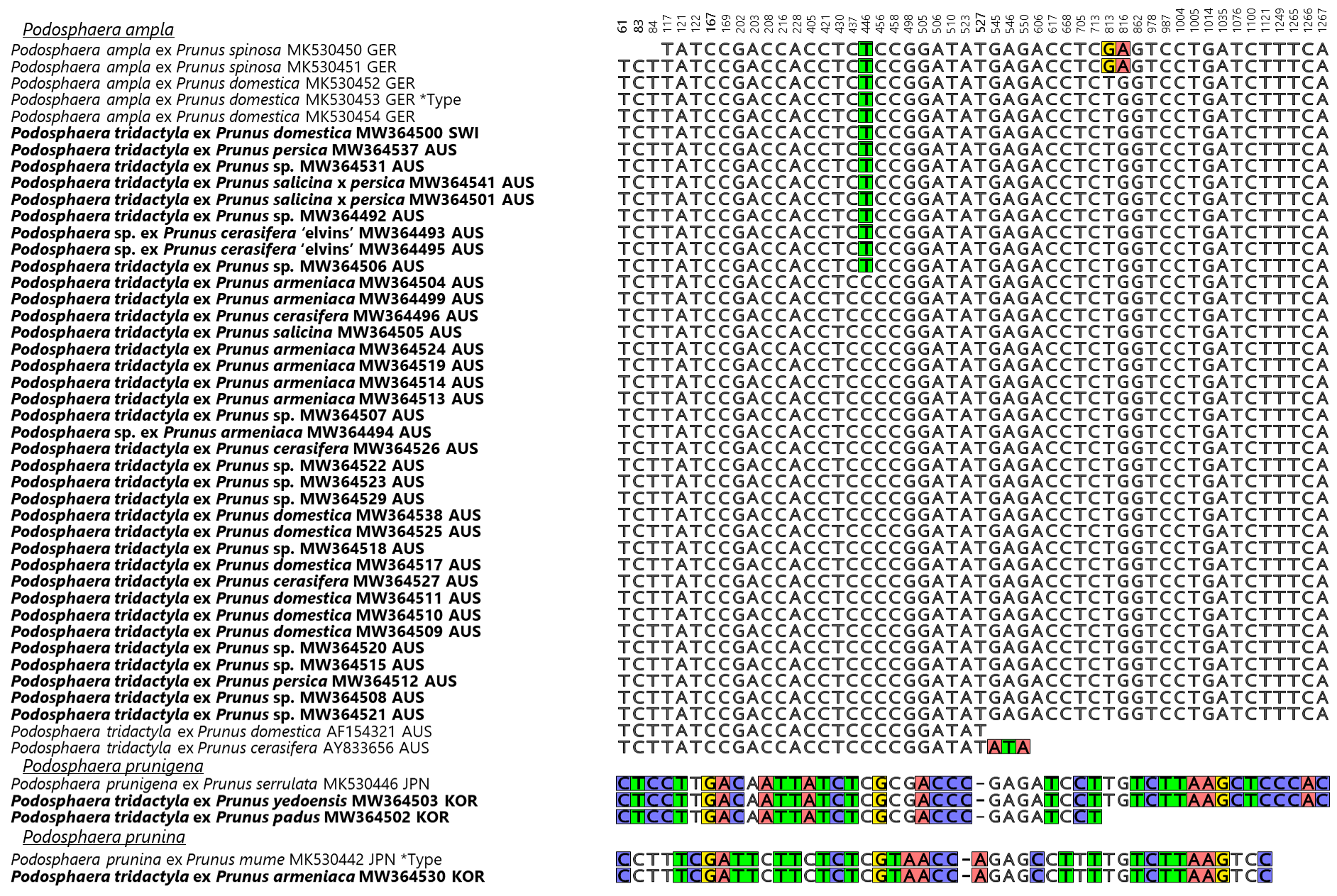


**Figure S1.** Summary of variable sites for *Podosphaera* *ampla* and sister taxa *Po*. *prunina* and *Po*. *prunigena*. Names on sequences are as deposited in reference collections prior to reidentification. * indicates type sequences. Sequences generated in this study are shown in bold and sequence names not in bold are from Meeboon et al [11]. from Numbers above the bases indicate base pair positions in the original alignment.
