# Supplementary material for: Re-Evaluation of the Podosphaera tridactyla Species Complex in Australia
Source: J Fungi (Basel). 2021 Feb 26;7(3):171. doi: 10.3390/jof7030171 (PMC8025908; doi:10.3390/jof7030171)
Supplement: Supplementary file 1 [file jof-07-00171-s001.zip › Figure S2.docx]

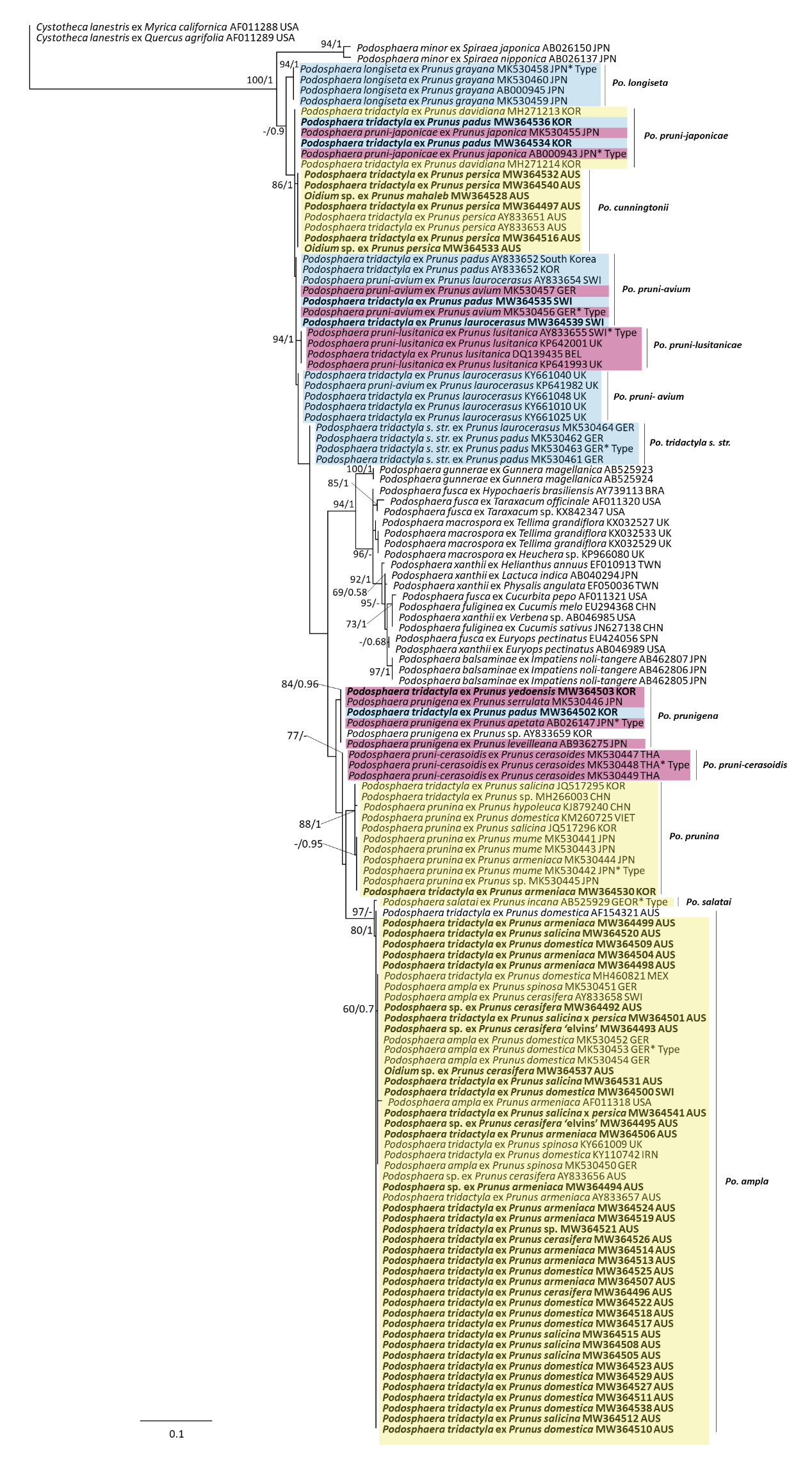


**Figure S2.** Maximum likelihood phylogenetic analysis of nuclear rDNA ITS1-5.8S-ITS2 sequences for the *Po*. *tridactyla* species complex and closely related species. Branch support values for maximum likelihood and Bayesian Inference analyses are shown when > 70% and 0.85 respectively. *Type indicates sequences obtained from isotype or holotype specimen for that species. Sequences generated in this study are shown in bold. Colours represent *Prunus* subgenera; *Cerasus* is pink, *Padus* is blue and *Prunus* is yellow.
