# Supplementary material for: Re-Evaluation of the Podosphaera tridactyla Species Complex in Australia
Source: J Fungi (Basel). 2021 Feb 26;7(3):171. doi: 10.3390/jof7030171 (PMC8025908; doi:10.3390/jof7030171)
Supplement: Supplementary file 1 [file jof-07-00171-s001.zip › Figure S4.docx]

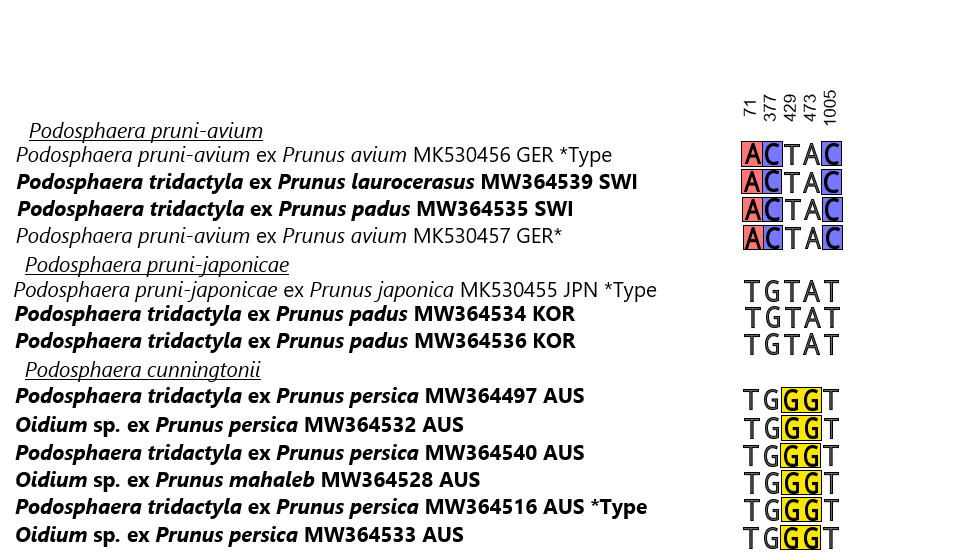


**Figure S4.** Summary of variable sites for *Po*. *cunningtonii* sequences compared to sister taxa *Podosphaera* *pruni-avium* and *Po. pruni-japonicae*. Names are as on sequences as deposited in reference collections prior to reidentification. * indicates type sequences from Meeboon et al [11]. Sequences generated in this study are shown in bold. Numbers above the bases indicate base pair positions in the original alignment.
